# Supplementary material for: A Likelihood Approach for Real-Time Calibration of Stochastic Compartmental Epidemic Models
Source: PLoS Comput Biol. 2017 Jan 17;13(1):e1005257. doi: 10.1371/journal.pcbi.1005257 (PMC5240920; doi:10.1371/journal.pcbi.1005257)
Supplement: S1 File — (TAR.GZ) [file pcbi.1005257.s014.tar.gz › HSPH_Online-SI-Revision/IPoi09/n100-severe/n100-severe_BENCH2_summary.pdf]

peak −8	3w spec	3w cum	5w spec	5w cum	end	3w spec LOG	3w cum LOG	5w spec LOG	5w cum LOG	end LOG
mean	388.1	385.	125.2	212.4	35.6	0.8	0.8	0.7	0.8	0.5
median	179.2	190.2	85.1	132.4	35.8	0.6	0.6	0.4	0.5	0.2
quantile .1	65.2	66.3	31.4	58.2	9.6	0.3	0.4	0.2	0.3	0.1
quantile .9	907.1	756.9	266.8	451.7	48.8	1.6	1.6	1.4	0.3	1.1
33	35	32	33	21						

peak −4	3w spec	3w cum	5w spec	5w cum	end	3w spec LOG	3w cum LOG	5w spec LOG	5w cum LOG	end LOG
mean	33.1	50.	41.3	25.3	30.1	0.2	0.2	0.3	0.1	0.1
median	32.	46.	39.4	25.9	26.7	0.2	0.2	0.2	0.1	0.1
quantile .1	23.	33.5	19.5	10.3	14.1	0.1	0.1	0.1	0.	0.1
quantile .9	43.1	67.4	68.1	35.	51.1	0.3	0.3	0.6	0.	0.2
47	38	38	40	16						

peak	3w spec	3w cum	5w spec	5w cum	end	3w spec LOG	3w cum LOG	5w spec LOG	5w cum LOG	end LOG
mean	80.1	48.6	169.5	59.8	78.9	0.2	0.2	0.4	0.2	0.2
median	65.9	42.1	140.9	52.8	70.7	0.2	0.1	0.3	0.2	0.2
quantile .1	36.4	27.	48.4	31.5	41.4	0.1	0.1	0.2	0.1	0.1
quantile .9	133.9	74.4	329.	93.	121.2	0.4	0.2	0.6	0.1	0.3
22	25	14	21	14						

peak +4	3w spec	3w cum	5w spec	5w cum	end	3w spec LOG	3w cum LOG	5w spec LOG	5w cum LOG	end LOG
mean	297.6	192.6	934.2	215.7	245.	0.5	0.4	0.6	0.4	0.4
median	245.9	178.1	349.2	197.4	234.9	0.5	0.4	0.6	0.4	0.5
quantile .1	78.4	65.	101.3	68.	86.5	0.2	0.2	0.2	0.2	0.2
quantile .9	446.5	307.7	870.7	323.1	384.9	0.6	0.5	0.9	0.2	0.6
11	12	8	11	11						

peak +8	3w spec	3w cum	5w spec	5w cum	end	3w spec LOG	3w cum LOG	5w spec LOG	5w cum LOG	end LOG
mean	7103.5	896.1	3680.8	1014.	1096.6	0.8	0.6	0.9	0.6	0.6
median	422.	345.3	554.9	365.7	406.1	0.7	0.6	0.7	0.6	0.6
quantile .1	97.5	96.3	121.6	100.1	105.	0.2	0.2	0.3	0.2	0.3
quantile .9	1233.3	817.1	12 185.7	938.1	984.4	1.1	0.9	2.	0.2	0.9
7	7	8	7	8	{}	{}	{}	{}	{}	{}
